# Continuous Gum Dentures

**Published:** 1888-12

**Authors:** W. H. Miller

**Affiliations:** Canton, Ohio


### Continuous Gum Dentures.

BY W. H. MILLER, D.D.S., CANTON, OHIO.

"It is a matter of regret that this, the most perfect method and style of constructing artificial teeth, should receive so little attention, and be employed so seldom it it is."

The above quotation is from on editorial in the DENTAL REGISTER, and it doubtless expresses a sentiment that every practitioner that believes in doing a good thing will respond to with a hearty Amen! Still the "Slaughter of the innocents" goes right along and the "store tooth" man finally gets in a set of warranted (?) teeth on a rubber plate using the sectional blocks, and frequently the only means that one would have of knowing that they were intended to represent natural teeth, was that they had been warranted to be teeth.

This continues to be the kind of dentures that are generally used, and the man who makes a set of teeth cheap can do as well as the one who charges a larger price. He can buy from the dealer on the same terms and his facilities for good mechanical results are frequently better than are found in the laboratory of the average *operative* dentist, who rather looks with disdain on *mechanical* dentistry in general, hence his products are fully equal to that of his nobler (?) competitor, while his price is less. The consequence is that the tendency has been towards the cheap.

No man has been able to offer anything that was worth more—considered artistically—than the cheap, until you reach the Continuous Gum Method. But here the price demanded is what constitutes the bar to its general introduction, and the great gap that exists between the operative and the mechanical in the profession is ever widening, without any apparent effort being made to restore the laboratory to the honored position it should justly hold in the minds of the profession.

In common with others I had felt that something might be done and should be done, that would give to the dentist the ability to produce dentures that should have all the advantages of *cheapness*, *strength*, and ARTISTIC RESULTS, and as nothing equals the continuous gum method for *artistic results*, the problem resolves itself to this: What can be done with continuous gum so that artistic results can be had, and yet compete in price with the cheap class of work that is flooding the country.

The method I am using in my practice and which seems to fill the requirements in all these particulars, is as follows: After having secured a model from the impression, make a die and counter-die of the gum and edge of the alveolar ridge, as far back as to include bicuspids, on this strike up a platina plate, this will now fit on the model, set up continuous gum teeth, back, solder, add body and enamel and fuse, now you have a block of the eight anterior teeth that is one solid piece, that having been made for the particular case, suits that case exactly, in all particulars, *strength*, *contour*, *thickness*, *and height* of the gum and the ARRANGEMENT OF THE TEETH. This block being now placed on the model in the articulator, the remaining teeth are set up in wax, plate waxed-up and vulcanized—using pink rubber for the gum from bicuspids to rear of plate, and finished in the ordinary way you now have a denture that *meets all the requirements* that can be asked for in a denture, *artistic*, *strong*, *light* and *cheap*. The expense does not materially exceed that of a set of sectional block teeth, and but little more labor, and there is a satisfaction in knowing that you have done the right thing and that you have the means within yourself of being able to do so every time, you can get a better price, with less trouble than for sectional block teeth. You have no joints to grind, no "dark joints" to fear, having once adopted this method and made yourself as familiar with the details as you are with the sectional block work, you will find that it will be rare that you will need or want the sectional block teeth, certainly never when you want to do the *very best*. This work can be attached to metal plates with the same ease as when sectional block teeth are used.

When this work is generally adopted, then the editor instead of regrets that this "the most perfect method and style of constructing artificial teeth should receive so little attention and be employed so seldom as it is" will have reason to rejoice that this method and style has been adopted and that the artistic in dentistry is appreciated and cultivated, and put into daily practice by all who would wish to be regarded as worthy and capable practitioners of dentistry.

